# Understanding the genetic and epigenetic architecture in complex network of rice flowering pathways

**DOI:** 10.1007/s13238-014-0068-6

**Published:** 2014-08-08

**Authors:** Changhui Sun, Dan Chen, Jun Fang, Pingrong Wang, Xiaojian Deng, Chengcai Chu

**Affiliations:** 1 Rice Research Institute, Sichuan Agricultural University, Chengdu, 611130 China; 2 State Key Laboratory of Plant Genomics and Center for Plant Gene Research (Beijing), Institute of Genetics and Developmental Biology, Chinese Academy of Sciences, Beijing, 100101 China

**Keywords:** rice, flowering time, genetic network, chromatin modifications, Arabidopsis, florigen

## Abstract

Although the molecular basis of flowering time control is well dissected in the long day (LD) plant Arabidopsis, it is still largely unknown in the short day (SD) plant rice. Rice flowering time (heading date) is an important agronomic trait for season adaption and grain yield, which is affected by both genetic and environmental factors. During the last decade, as the nature of florigen was identified, notable progress has been made on exploration how florigen gene expression is genetically controlled. In Arabidopsis expression of certain key flowering integrators such as *FLOWERING LOCUS C* (*FLC*) and *FLOWERING LOCUS T* (*FT*) are also epigenetically regulated by various chromatin modifications, however, very little is known in rice on this aspect until very recently. This review summarized the advances of both genetic networks and chromatin modifications in rice flowering time control, attempting to give a complete view of the genetic and epigenetic architecture in complex network of rice flowering pathways.

## INTRODUCTION

Rice flowering time (heading date), which is affected by both endogenous and exogenous factors, is an important agronomic trait for regional and seasonal adaption. Heading on a proper time is the most critical step for grain production. Precocious flowering reduces the vegetative phase and leads to reduction of biological yield. On the other hand, delayed flowering could cause low seed setting percentage in cold late autumn or delay next planting season, which both results in production loss.

**Figure 1:**
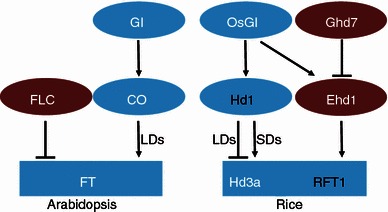
**Comparison of core-flowering-pathways in rice and Arabidopsis**. *OsGI*/*GI*-*Hd1*/*CO*-*Hd3a*/*FT* pathway is conserved between rice and Arabidopsis. *CO*. accelerates flowering under LD, however, *Hd1* promotes flowering under SD and represses it under LD. Besides, *Ghd7* and *Ehd1* in rice, and *FLC* in Arabidopsis are unique flowering integrators, respectively. *FLC* and *Ghd7* are major flowering suppressors, while *Ehd1* acts as a flowering promoter

Florigen is produced in the leaf under inductive day length conditions and transported to the shoot apex where it triggers flowering transition (Cajlachjan, 1937; Corbesier et al., 2007; Tamaki et al., 2007). Unlike only one florigen gene *FLOWERING LOCUS T* (*FT*) in Arabidopsis, rice evolves two florigen genes, *Heading date 3a* (*Hd3a*) and *RICE FLOWERING LOCUS T 1* (*RFT1*), and at least two flowering pathways are developed to control the expression of florigens, the *Heading date 1* (*Hd1*) pathway which is conserved between rice and Arabidopsis, and the *Early heading date 1* (*Ehd1*) pathway which is unique to rice (Doi et al., 2004) (Fig. 1). Numerous studies reveal that a large number of rice genes regulate flowering time through the two flowering integrators.

In Arabidopsis, some flowering regulators such as *FLC* and *FT* are reported to be regulated by various chromatin modifications (He, 2009; Liu et al., 2010). However, little is known in rice in this field. Recently, we characterized a major histone methyltransferase (HMTase) gene *SET DOMAIN GENE 724* (*SDG724*), which is required for Histone H3 lysine 36 (H3K36) methylation, promotes rice heading, indicating that rice flowering could also be regulated by chromatin modifications (Sun et al., 2012). In the past two years, more and more molecular genetic studies gave the clues on the chromatin modification mechanism in rice flowering pathways, we summarize here most recent advances towards understanding of genetic networks and epigenetic chromatin modifications in rice flowering time control.

## TWO FLORIGEN GENES *Hd3a* AND *RFT1* IN RICE

Florigen, which has been hypothesized by many physiological studies, is believed to be produced in leaves by the inductive photoperiod, then moves to the shoot apical meristem (SAM) and triggers flowering transition. But this florigen has been eluded identification since it was first proposed for 70 years (Cajlachjan, 1937). In 2007, it was firstly revealed that *FT* encoded protein in Arabidopsis, is a leaf-derived long-distance signal directed to floral transition (Corbesier et al., 2007).

In rice, there are 13 *FT* homologs in the genome (Chardon and Damerval, 2005), *Hd3a* and *RFT1* are two of them which were confirmed to act as florigen genes (Komiya et al., 2008; Komiya et al., 2009; Tamaki et al., 2007). By fusing *Hd3a* or *RFT1* with GFP, it was demonstrated that Hd3a or RFT1 protein was expressed in vascular tissue of leaves, and could be moved to SAM where they started flowering induction. As Hd3a-GFP was only detected in the SAM of plants grown under short day conditions (SD), RFT1-GFP was merely detected under long day conditions (LD) (Komiya et al., 2009; Tamaki et al., 2007). On the other hand, *Hd3a-RNAi* (*RNA interference*) plants significantly delayed heading date under SD but not LD, *RFT1-RNAi* plants flowering was obviously delayed under LD but not SD oppositely. Furthermore, rice with knockout of both Hd3a and RFT1 caused at least 300 days late flowering under both SD and LD (Komiya et al., 2009). All these data demonstrated that, unlike Arabidopsis, rice has two florigen genes, *Hd3a* and *RFT1*, *Hd3a* is responsible for flowering under inductive SD, whereas *RFT1* is responsible for flowering transition under non-inductive LD. Although *Hd3a* and *RFT1* are located in some chromosome and separated by only 11.5 kb in the genome, the fine-tuning of long day flowering by the H3K36me2/3 level of *RFT1* but not *Hd3a* via SDG724, therefore, *RFT1* and *Hd3a* which have functionally diverged to control flowering time under LD and SD conditions are partly due to a fine-tuned epigenetic mechanism (Sun et al., 2012).

## FLORIGEN REGULATED NETWORK

How flowering pathways are regulated differs in plants. In Arabidopsis, flowering is controlled by a small number of large-effect genes such as *FLC* (Salome et al., 2011), whereas in maize is controlled by many additive small-effect quantitative trait loci (QTLs) (Buckler et al., 2009). Interestingly, rice combines both regulatory manners, including a few large-effect factors, such as *Hd1*, *Ehd1*, and *Grain number*, *plant height andheading date 7* (*Ghd7*), in addition to some small-effect QTLs and genes (Ebana et al., 2011; Tsuji et al., 2013) (Table 1).

**Table 1:**
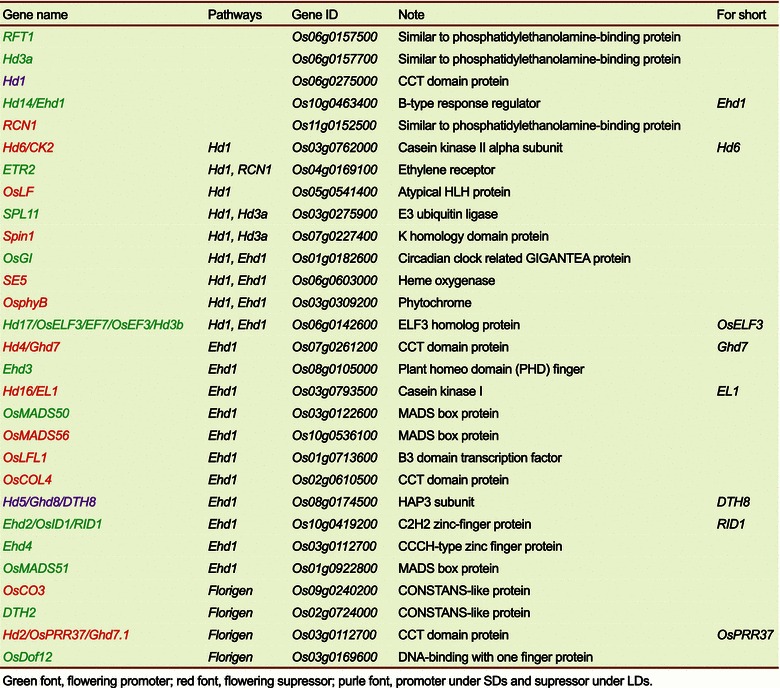
Flowering regulators in rice

**Figure 2:**
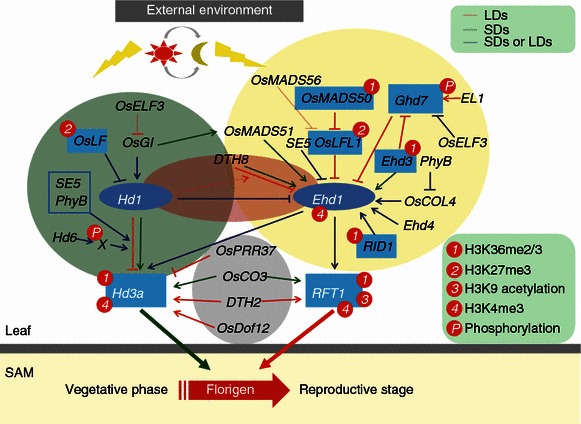
**Complex flowering time control network in rice**. Rice flowering network is formed by two florigen genes *Hd3a* and *RFT1*, and four regulation modules, including *Hd1*-dependent pathway, *Ehd1*-dependent pathway, crosstalk between *Hd1* and *Ehd1* pathway, and flowering regulators independent of *Hd1* and *Ehd1*. The first three signals come together to regulate *Hd1* and *Ehd1* and affect florigen gene expression; the last module may directly control the expression of florigen genes independent of *Hd1* and *Ehd1*. Besides, expressions of *Ehd3*, *RID1*, *OsMADS50*, *Hd3a*, and *RFT1* can be affected by H3K36me2/3; *OsLFL1* and *OsLF* transcriptions can be mediated by H3K27me3. Finally, all those florigen signals are transported from leaves to SAM and trigger flowering transition there. All the gene names for short are showed in Table 1

So far, quite a number of QTLs controlling rice heading date (Hd) were identified and characterized using different segregating populations derived from crossing a japonica cultivar (*Nipponbare*) and an indica cultivar (*Kasalath*) (Lin et al., 1998; Yano et al., 1997). These QTLs include the major loci controlling photoperiodic flowering responses, *Hd1* (Yano et al., 1997; Yano et al., 2000), *Hd2*/*Ghd7.1*/*OsPRR37* (*Oryza sativa Pseudo*-*Response Regulator 37*) (Koo et al., 2013; Liu et al., 2013; Shibaya et al., 2011; Yamamoto et al., 2000; Yan et al., 2013), *Hd3a* (Kojima et al., 2002), *Hd4*/*Ghd7* (*Ghd7* for short) (Koo et al., 2013; Xue et al., 2008), *Hd5*/*Days to heading 8*/*Grain number*, *Plant height*, *and Heading date 8*/*Late Heading Date 1* (*Hd5*/*DTH8*/*Ghd8*/*LHD1*) (Dai et al., 2012; Fujino et al., 2013; Yan et al., 2011). Furthermore, backcross progenies derived from the same original cross allowed identification of other QTLs, such as *Hd6*/*CK2* (*CASEIN KINASE 2*) (Ogiso et al., 2010; Takahashi et al., 2001; Yamamoto et al., 2000), *Hd14*/*Ehd1* (Doi et al., 2004), *Hd16*/*EL1* (*Early flowering 1*) (Dai and Xue, 2010; Hori et al., 2013; Shibaya et al., 2011), *Hd17*/*OsELF3*/*EF7*/*OsEF3*/*Hd3b* (*Hd17*/*Oryza sativa Early Flowering 3*/*Early Flowering 7*/*Oryza sativa Early Flowering 3*/*Hd3b*, *OsELF3* for short) (Hori et al., 2012; Matsubara et al., 2012; Saito et al., 2012; Yang et al., 2013; Zhao et al., 2012). Additionally, using rice near isogenic lines and mutants, more genes implicated in controlling flowering time have been identified and positioned into a regulatory network (Brambilla and Fornara, 2013; Itoh and Izawa, 2013; Tsuji et al., 2011, 2013) (Fig. 2).

## *Hd1*-DEPENDENT PATHWAY

There is a similar molecular system for florigen control in Arabidopsis and rice (Izawa, 2007; Tsuji et al., 2011). *Hd1* and *Hd3a* in rice are homologs of *CONSTANS* (*CO*) and *FT* in Arabidopsis, respectively. As in Arabidopsis, *Hd1* acts upstream of *Hd3a* (Kojima et al., 2002; Yano et al., 2000), and overexpression of a rice ortholog of Arabidopsis *GIGANTEA* (*GI*) which acts upstream of *CO*, namely *OsGI*, increased the expression of *Hd1* in the transgenic plants, followed by suppressing *Hd3a* expression, resulting in late flowering under both SD and LD (Hayama et al., 2003). Differently, *CO* merely promotes *FT* expression, *Hd1* plays a more enigmatic role in rice, which promotes flowering under SD, but represses flowering under LD (Hayama et al., 2003; Komiya et al., 2008; Lin et al., 2000; Tamaki et al., 2007). These results indicate that the core photoperiodic pathway composed of the three key flowering genes *OsGI*/*GI*-*Hd1*/*CO*-*Hd3a*/*FT* is conserved between rice and Arabidopsis, but its function has diverged during evolution to produce opposite flowering responses. While the photoperiodic pathway in Arabidopsis merely accelerates flowering under LD, in rice, it promotes flowering under SD and represses flowering under LD (Takahashi and Shimamoto, 2011).

The reversible mechanism that *Hd1* functions as either an activator or suppressor of *Hd3a* involves the action of the red-light photoreceptor phytochrome B (phyB), since mutations in *phyB* or phytochrome chromophore synthesis, such as *photoperiod sensitivity 5* (*se5*), attenuate this conversion and maintain *Hd1* as an activator under any photoperiodic conditions. On the other hand, though *Hd1*-overexpressing plants delay flowering, Hd1 protein levels in these plants are not significantly altered (Andres et al., 2009; Ishikawa et al., 2011; Izawa et al., 2002), thus it is speculated that LD light signals may modify the protein of Hd1 or Hd1 complex through phytochrome but not its expression levels, and convert it into a repressor of flowering. Therefore, uncovering of the biochemical function of Hd1 protein and the molecular nature of its dual activity will provide exciting insight into the control of photoperiodic flowering in rice.

Recently, it is deduced that Hd1 protein activity is possible affected by an additional posttranslational regulatory factor, *Hd6*, which encodes a CK2 α-subunit (Ogiso et al., 2010; Takahashi et al., 2001). The delay flowering effect of Hd6 is observed only when Hd1 is functional, however, Hd1 is not phosphorylated by Hd6 *in vitro* (Ogiso et al., 2010), suggesting that Hd6 phosphorylates unknown substrates that cooperate with Hd1 in the LD floral suppression pathway.

## *Ehd1* DEPENDENT PATHWAY

In 2004, a novel regulatory *Ehd1-*pathway which is not presented in Arabidopsis, is discovered in rice (Doi et al., 2004). *Ehd1*, encoding a B-type response regulator, is a floral promoter, and rice variety Taichung 65 (T65) without functional *Ehd1* allele delays flowering under both LD and SD (Doi et al., 2004). As it has been shown that *Ehd1* contributes to flowering time by its expression levels (Takahashi et al., 2009), thus fine-tuning of *Ehd1* expression is crucial for rice flowering at suitable time, and several flowering regulators have been identified to participate in this regulation.

*Ghd7*, which is important for increasing rice productivity and adaptability, is a major regulator of *Ehd1* and could delay flowering by repressing *Ehd1* under LD (Takahashi et al., 2009; Xue et al., 2008). As *Ghd7* encodes a CCT (CONSTANS, CO-like, and TOC1) domain protein, which shows very low homology to Arabidopsis genome, the *Ghd7*-*Ehd1* may be a unique pathway in rice (Koo et al., 2013; Xue et al., 2008). Further study shows that *Ghd7* and *Ehd1* can respectively set a daylength threshold for *Hd3a* expression, which is usually observed in SD plants but not in LD plants (Itoh et al., 2010; Takimoto and Ikeda, 1961), and this capacity of discernment in critical day length in rice greatly enriches the daylength-dependent regulated mechanism of florigen gene expression.

Until now, at least three genes, *Early heading date 3* (*Ehd3*), *ELF3*, and *Hd16*/*EL1*, were identified to control *Ghd7* expression in *Ehd1-*pathway. *Ehd3* encodes a plant homeodomain (PHD) finger protein and is identified as one repressor of *Ghd7*. Generally, *Ghd7* transcript reaches its highest level after seeding for two weeks, and then the expression is gradually reduced to a basal level, but in *ehd3* mutants, *Ghd7* expression level is always higher and delays heading date for more than one year under LD. Interestingly, under SD, *Ehd3* could promote *Ehd1* expression regardless of *Ghd7*, suggesting a perplexed role of *Ehd3* (Matsubara et al., 2011).

*ELF3* in Arabidopsis is responsible for generating circadian rhythm and regulating photoperiodic flowering, consistently, its homolog in rice *OsELF3* is also required to sustain the robust oscillation, and lesions in *OsELF3* delay flowering under both SD and LD (Saito et al., 2012; Yang et al., 2013; Zhao et al., 2012). Under SD, *OsELF3* promotes flowering mainly by repressing *Ghd7*, because late flowering of *oself3* mutants can be rescued if *Ghd7* but not *Hd1* is mutated. Under LD, *oself3* mutants increase *OsGI* and *Ghd7* expression, thus up-regulate *Hd1* and repress *Ehd1* expression, respectively, indicating that *OsELF3* influences photoperiodic flowering in both *Hd1* and *Ehd1* pathways (Brambilla and Fornara, 2013; Saito et al., 2012).

*Hd16*/*EL1*, encoding a casein kinase I protein, is associated with the gibberellin-mediated flowering transition (Dai and Xue, 2010). Deficient in *Hd16* weakens rice photoperiod sensitivity, but increases *Ehd1*, *Hd3a*, and *RFT1* expression under LD. Though the expression level of *Ghd7* is not significantly altered in *el1* mutants, the biochemical data indicate that *Hd16* acts as a flowering repressor by phosphorylation of *Ghd7* (Dai and Xue, 2010; Kwon et al., 2013).

*OsLFL1* (*Oryza sativa LEC2 and FUSCA3 Like 1*) encodes a putative B3 transcription factor, knockdown of *OsLFL1* does not affect flowering time, while ectopic overexpression of *OsLFL1* decreases *Ehd1* expression and results in late flowering (Peng et al. 2007, 2008). *OsLFL1* is controlled by two members of MIKC-type MADS-box family, *OsMADS50* and *OsMADS56*. Both *osmads50* mutants and *OsMADS56*-overexpressing plants, which produce increased *OsLFL1* expression, show late flowering phenotype (Lee et al., 2004; Ryu et al., 2009). Interestingly, OsMADS56 can interact with OsMADS50 *in vitro*, suggesting that the two MADS-box members tend to form a heterodimer complex and function antagonistically through *OsLFL1*-*Ehd1* pathway under LD (Ryu et al., 2009).

As mentioned in *Hd1*-pathway, phytochrome is probably a primary cause of *Hd1-*dependent suppression of rice flowering, but underlying molecular mechanism of phytochrome in *Ehd1*-pathway is not well understood. Recent studies showed that *SE5* and *phyB* also suppress *Ehd1* expression, and the *phyB*-mediated suppression of *Ehd1* is confirmed to be repressed by a CONSTANS-like (COL) gene *OsCOL4* (*Oryza sativaCONSTANS-like4*) (Andres et al., 2009; Komiya et al., 2009; Lee et al., 2010). *OsCOL4* expression is decreased in *osphyB* mutants, and *osphyB oscol4* double mutants flower is similar to *osphyB* single mutants, indicating that *OsCOL4* functions downstream of *OsphyB* (Lee et al., 2010).

Besides the above regulators, *Ehd1* expression is also modulated by other four flowering factors independently. *Indeterminate 1* (*ID1*) is one of them, which expresses in leaf but induces flowering in the shoot meristem. *ID1* has been once thought to be involved in the florigen synthesis in maize (Colasanti et al., 2006; Colasanti et al., 1998), and its regulated mechanism has been exhibited in rice. Lesions in rice *RID1* (*Early heading date 2*/*OsINDETERMINATE 1*/*Rice INDETERMINATE 1*, *Ehd2*/*OsID1*/*RID1, RID1* for short) lead to extremely late flowering phenotype, as well as decreased expression of *Ehd1* and downstream florigen genes under both SD and LD (Matsubara et al., 2008; Park et al., 2008; Wu et al., 2008).

*Ehd4* (*Early heading date 4*), encoding a CCCH-type zinc finger transcriptional regulator, is expressed mostly in immature leaves and shows a similar diurnal expression pattern of *Ehd1* under both SD and LD. *Ehd4* up-regulates the expression of the florigen genes *Hd3a* and *RFT1* through *Ehd1*. Strikingly, *Ehd4* is highly conserved in both wild rice and cultivated rice, but homologs cannot be found in other species, suggesting that *Ehd4* is unique flowering regulator in *Oryza* genus differed from other grass members during evolution (Gao et al., 2013).

*OsMADS51* is another MADS box gene, other than *OsMADS50* and *OsMADS56*, it acts downstream of *OsGI*, transmits a promotion signal from *OsGI* to *Ehd1* under SD. Though its null mutants showed late flowering phenotype followed by decreased expression of *Ehd1* and *Hd3a*, ectopic expression of *OsMADS51* causes early flowering, accompanying with increased expression *Ehd1* and *Hd3a* (Kim et al., 2007).

*Hd5*/*DTH8*/*Ghd8*/*LHD1* encodes a putative HEMEACTIVATOR PROTEIN 3 (HAP3) subunit of a CCAAT-box binding protein (HAP complex) that binds to CCAAT boxes in yeast and animals. Similar to *Hd1*, *Hd5*/*DTH8*/*Ghd8*/*LHD1* delayed flowering in rice under LD and promotes flowering under SD, but by regulating expression of *Ehd1* (Dai et al., 2012; Lin et al., 2003; Wei et al., 2010; Yan et al., 2011).

Most interestingly, **t**hough *Hd5*/*DTH8*/*Ghd8*/*LHD1* suppresses rice heading though *Ehd1*, genetic analysis implies that *Hd5* requires functional *Hd1* to repress flowering under LD (Nonoue et al., 2008), rising a question what is the relationship between *Hd1* and *Ehd1*. Recent findings indicate that transcript level of *Ehd1* is down-regulated in *Hd1-*overexpression transgenic lines, suggesting that, to some degree, *Hd1* is an upstream regulator of *Ehd1* expression, but how this crosstalk works is still undefined (Ishikawa et al., 2011).

## FLOWERING REGULATORS INDEPENDENT OF *Hd1* AND *Ehd1*

Besides *Ehd1*, T65 also bears a loss-of-function allele of *Hd1*, but it could still flower in time and serves as a commercial rice variety, so there are must some other regulators independent of *Hd1* and *Ehd1* in rice flowering network (Doi et al., 2004). *OsCO3* and *DTH2* are two of them, and promote flowering by regulating florigen genes. Though both of them are COL genes, they function under different photoperiodic conditions. Expressions of *Hd3a* and *FT-like* genes are decreased in the *OsCO3-*overexpressing plants under SD without altered expression of other florigen upstream regulators, suggesting that *OsCO3* primarily controls flowering time under SD by negatively regulating the expression of florigen genes, independent of other known SD-promotion pathways (Kim et al., 2008). For *DTH2*, both association analysis and transgenic experiments indicate that two functional nucleotide polymorphisms that correlated with early heading and increased reproductive fitness under natural LD in northern Asia. Further combined population genetics and network analyses suggest that *DTH2* probably represents a target of artificial selection for adaptation to LD during rice domestication and improvement, demonstrating an important role of minor effect quantitative trait loci in crop adaptation and breeding (Wu et al., 2013).

Although some *PRR* genes are major components of the circadian oscillator, a rice *PRR* gene *Hd2*/*Ghd7.1*/*OsPRR37* may down-regulate *Hd3a* expression independent of any known pathways to suppress flowering under LD. As lesions in *Hd2*/*Ghd7.1*/*OsPRR37* cause early flowering phenotype, the japonica varieties harboring nonfunctional alleles of both *Ghd7* and *Hd2*/*Ghd7.1*/*OsPRR37* flower extremely early under natural LD, and make these varieties adapt to the northernmost rice cultivation regions. Further study implied that natural variations in *Hd2*/*Ghd7.1*/*OsPRR37* have contributed to the expansion of rice cultivation to temperate and cooler regions (Koo et al., 2013; Liu et al., 2013; Yan et al., 2013).

Different from *Hd2*/*Ghd7.1*/*OsPRR37*, *OsDof12* is LD-specific flowering repressor and encodes a DNA-binding with one finger (Dof) transcription factor which is involved in a variety of biological processes of plants. The transcriptions of *OsDof12* can express at different development stages, but strongly inhibited by dark treatment. *OsDof12*-overexpressing plants flower earlier in consistent with the up-regulation of *Hd3a* independent of other flowering genes under LD but not SD (Li et al., 2009).

## CHROMATIN MODIFICATIONS REGULATE FLOWERING IN RICE

Chromatin, which is composed by complexing DNA with histone, carries not only genetic, but also epigenetic information. In Arabidopsis, the expression of a major flowering repressor *FLC* is regulated by a number of active and repressive chromatin modifications, such as histone tails methylation, acetylation, ubiquitination etc. In addition, histone modifications can also directly regulate the expression of florigen gene *FT*, and the regulation manner of *FLC* and *FT* provides a paradigm for control of developmental regulators through chromatin modifications (He, 2009). Currently, not so many data are available about that in rice, but molecular genetic studies indicated that rice flowering control also undergoes the complex chromatin modifications (Table 2).

**Table 2:** Chromatin modification regulators in rice

Gene name	Pathways	Gene ID	Note	Modifications	Target genes
*SDG724*	*Ehd1*	*Os09g0307800*	SET domain group protein	H3K36me2/3	*RFT1*, *OsMADS50*
*SDG725*	*Ehd1*	*Os02g0554000*	SET domain group protein	H3K36me2/3	*Ehd3, Ehd2, OsMADS50,Hd3a, RFT1*
*LC2*/*OsVIL3*	*Hd1*	*Os02g0152500*	Plant homeo domain (PHD) finger	H3K27me3	*OsLF*
*OsVIL2*	*Ehd1*	*Os12g0533500*	Plant homeo domain (PHD) finger	H3K27me3	*OsLFL1*
*OsEMF2b*	*Ehd1*	*Os04g0162100*	C2H2 zinc-finger protein, interact with *OsVIL2*	H3K27me3	*OsLFL1*
*OsTrx1*	*Ehd1*	*Os09g0134500*	SET domain group protein, interact with *Ehd3*	Unkown	Unkown

## ACTIVE CHROMATIN MODIFICATIONS AND RICE FLOWERING

S-Adenosyl-l-methionine is a universal methyl group donor involved in numerous transmethylation reactions, including histone methylation. Knockdown of rice *S-Adenosyl-l-methionine synthetase* (*SAMS*) *1*, *2*, and *3* greatly reduced the expression of *Ehd1*, *Hd3a*, *RFT1* and led to a late flowering phenotype. Moreover, the histone H3K4me3 and symmetric DNA methylation at these genes was significantly reduced, suggesting an association between epigenetic modification and flowering in rice, but more research are required on this relationship (Li et al., 2011).

We have demonstrated that *SDG724*, a histone methyltransferase gene which belongs to SET domain family Class II (Ng et al., 2007), affected flowering time by mediating H3K36 methylation in rice. *SDG724* loss-of-function mutant *lvp1* showed a late flowering phenotype under both LD and SD, which was associated with the suppressed expression of *RFT1* and *Hd3a*. Interestingly, only the chromosomal region of *RFT1*, but not *Hd3a*, reduced the level of H3K36me2/3 modifications which associated with the transcriptionally active chromatin state, although the two florigenic genes are closely linked in the genome and separated by only 11.5 kb (Sun et al., 2012). This similar regulated way in *RFT1* is also found in a previous report that *RFT1* expression can be promoted through another active histone modification H3K9 acetylation around the transcriptional start site of its chromatin in *Hd3a*-RNAi transgenic plants (Komiya et al., 2008). In conclusion, both of the two findings suggest an epigenetic regulation mechanism through *RFT1*. In addition, *SDG724* also affects the histone modification state at *OsMADS50* chromosomal region, thus all the results suggest a LD floral promotion pathway mediated by H3K36me2/3 deposition through *OsMADS50*-*Ehd1*-*RFT1* pathways in rice (Sun et al., 2012).

Coincidentally, another member of Class II in SET domain family (Ng et al., 2007), *SDG725*, is also involved in promoting rice flowering through H3K36me2/3. In *SDG725* knockdown plants, the expression levels of *Ehd3*, *RID1*, *OsMADS50*, *OsMADS51*, *Ehd1*, *Hd3a*, and *RFT1* were all drastically reduced, but the *Ghd7* expression was increased, under either SD or LD. Different from *SDG724*, *SDG725* is required for deposition of H3K36me2/3 at more flowering gene loci, such as *Ehd3*, *RID1*, *OsMADS50*, *Hd3a*, and *RFT1*. Thus, *SDG724* and *SDG725* regulate both overlapped and specific flowering genes by mediating H3K36me2/3 deposition and promote rice flowering, which are different to the previously known function of these epigenetic marks in Arabidopsis flowering (Sui et al., 2012; Xu et al., 2008; Zhao et al., 2005).

Very recently, another homolog of *SDG724*, *OsTrx1*, which belongs to SET domain family Class III (Ng et al., 2007), might activate or maintain the active transcribed states of target genes, and was reported to delay flowering time under LD through *Ghd7* pathway but not *OsMADS50* and *Hd1* pathways (Choi et al., 2014). Though expression of *Ehd3* that functions upstream of *Ghd7* is unchanged in *ostrx1* mutants, it was proved that OsTrx1 could bind to Ehd3 *in vitro*. Further study showed that PHD motif of OsTrx1 could bind to native histone H3 and the C-terminal end of SET domain of OsTrx1 has histone H3 methyltransferase activity, thus OsTrx1 and Ehd3 tend to form a complex to methylate downstream genes, but further studies are needed to illuminate its function in detail (Choi et al., 2014).

## REPRESSIVE CHROMATIN MODIFICATIONS AND RICE FLOWERING

Arabidopsis *VILs* (*VIL*, *VERNALIZATION INSENSITIVE*), *VIN3*, and *VRN5* are components of PRC2 (Polycomb Repressive Complex 2), mediating the H3K27 trimethylation at the *FLC* locus to repress its expression and hence to induce flowering. In rice, a *VIL* homolog gene *LC2*/*OsVIL3* is considered as a possible component of PRC2 complex, and *lc2* mutants display late flowering along with the reduced expression of *Hd1* and *Hd3a* under SD. Furthermore, consistent with the result that *OsLF* (*Oryza sativaLate Flowering*) directly repressed *Hd1* expression (Zhao et al., 2011), *LC2*/*OsVIL3* binds to the promoter region of *OsLF* and represses the *OsLF* expression via H3K27me3 methylation, thus eventually promotes flowering (Wang et al., 2012).

*OsVIL2* may be another *VILs* member in rice PRC2 complex, and mutations in *OsVIL2* cause late flowering under both SD and LD. Different from *LC2*/*OsVIL3*, the late flowering phenotype is associated with increased *OsLFL1* and reduced *Ehd1*, *Hd3a*, *RFT1* expression. Furthermore, OsVIL2 can bind to native histone H3 *in vitro* and is directly associated with *OsLFL1* chromatin *in vivo*, and H3K27me3 is significantly reduced on *OsLFL1* chromatin in *osvil2* mutants compared to the wild type, indicating that *OsVIL2* epigenetically represses *OsLFL1* expression to promote flowering in rice. Besides, OsVIL2 can physically interact with OsEMF2b, which may be also a member of PRC2. Similar to *osvil2*, a null mutation of *OsEMF2b* caused late flowering by increasing *OsLFL1* and decreasing *Ehd1* expression (Wang et al., 2012; Yang et al., 2012).

In short, similar to Arabidopsis, *LC2*/*OsVIL3*, *OsVIL2*, and *OsEMF2b* may function together with PRC2 to induce flowering by affecting histone modification H3K27me3, but their target flowering genes are different, indicating that a diverse flowering pathway regulated by PRC2 in rice flowering.

## CONCLUSION AND PERSPECTIVES

Heading date is an important agronomic trait that determining rice to grow in different regions and seasons. In last two decades, tremendous progress has been made by the study of QTLs and genes controlling rice flowering, which not only identified the nature of the mobile signal florigen, but also unveiled a complex genetic network that controls florigen in rice. Hd3a and RFT1, two florigens regulated respectively rice flowering under SD and LD, are mainly controlled through *Hd1* and *Ehd1* pathways. However, as mentioned in various T65 with lack of both, rice also develops some additional pathways that could induce rice flowering.

Histone modification is very important for defining transcriptional regulation expression, thus plays a fundamental role in plant growth and development, as well as responding to various environmental conditions. These modification marks are dynamically “written” and “erased”, and then specifically recognized by the “readers” and instruct specific biological process, such as flowering. Very recently, a large number of studies have revealed that various ‘active’ histone modifications, H3K4 methylation, H2B monoubiquitination, H3K36me2/me3, histone deacetylation, and ‘repressive’ chromatin modifications, H3K4 demethylation, H3K9 methylation, H3K27 methylation, histone arginine methylation, are involved in modulating *FLC* expression in Arabidopsis. Though the regulation of *FLC* expression via chromatin modification provides a paradigm in flowering gene expression, whether there exists a major flowering regulator such as *FLC* in rice is still unknown (Fig. 1). Rice possibly has some new routes in its flowering control. In rice, a number of studies revealed the difference in chromatin modification mechanism in the past two years, ‘active’ H3K36me2/3, H3K4me3, H3K9 acetylation and ‘repressive’ H3K27me3 modifications mediate flowering time through *Hd1* and *Ehd1* dependent pathways, and our finding about *SDG724* also suggests a LD floral promotion pathway that could be mediated via an epigenetic regulation of florigen *RFT1* itself. All these data suggest that the target flowering genes of chromatin modifications are dispersed in both conserved and unique flowering pathways in rice. Taken together, all the progress in rice, along with Arabidopsis, provides a complete evolutionarily comparative view of genetic and epigenetic flowering mechanisms in plants until now.

Furthermore, in rice, some histone modification participators tend to function under SD and LD, but others like to function mainly under SD or LD, thus unveiling of histone modification mechanism in rice flowering might set a solution to verify the relationships between particular histone modifications and photoperiod environments. On the other hand, as a LD plant, Arabidopsis flowering is accelerated by LD, but SD plant rice flowers earlier under SD than under LD, further study will be helpful to distinguish the function and evolutionary process of histone modification in various photoperiodic plants. Thus, it would be of great interest to identify more chromatin modification regulators and their target genes in rice flowering in future.

## ABBREVIATIONS

*FLC*: *FLOWERING LOCUS C*
*FT*: *FLOWERING LOCUS T*
H3K36: Histone H3 lysine 36
Hd: heading date
HMTase: histone methyltransferase
LD: long day
*RFT1*: *RICE FLOWERING LOCUS T 1*
SAM: shoot apical meristem
SD: short day
